# Preoperative lung immune prognostic index predicts survival in patients with pancreatic cancer undergoing radical resection

**DOI:** 10.3389/fsurg.2022.1002075

**Published:** 2023-01-06

**Authors:** Qian Zhou, Guochao Deng, Zhikuan Wang, Guanghai Dai

**Affiliations:** ^1^ Department of Oncology, Medical School of Chinese People’s Liberation Army (PLA), Beijing, China; ^2^ Department of Medical Oncology, The First Medical Center of Chinese PLA General Hospital, Beijing, China; ^3^ School of Medicine, Nankai University, Tianjin, China; ^4^ Department of Medical Oncology, The Fifth Medical Center of Chinese PLA General Hospital, Beijing, China

**Keywords:** pancreatic cancer, lung immune prognostic index, radical resection, prognosis, DNLR, LDH

## Abstract

**Background:**

Lung immune prognostic index (LIPI), a combination of derived neutrophil-to-lymphocyte ratio (dNLR) and lactate dehydrogenase (LDH), is currently attracting considerable interest as a potential prognostic indicator in many malignancies. Our study aimed to investigate the prognostic value of preoperative LIPI in patients with pancreatic ductal adenocarcinoma (PDAC) undergoing radical resection.

**Methods:**

We retrospectively reviewed PDAC patients treated with radical resection from February 2019 to April 2021 at Chinese People's Liberation Army (PLA) general hospital. Based on the cut-off value of dNLR and LDH identified by X-tile, patients were divided into LIPI good and LIPI intermediate/poor group. Kaplan-Meier curve and log-rank test were used to compare the recurrence-free survival (RFS) and overall survival (OS) of the two groups. Univariate and multivariate Cox regression was used to identify the independent prognostic value of LIPI. Subgroup analysis was performed to identify specific population benefited from radical resection.

**Results:**

A total of 205 patients were included and the median RFS and OS was 10.8 and 24.3 months, respectively. Preoperative LIPI intermediate/poor was related to worse RFS and OS (*p* < 0.05). Preoperative LIPI intermediate/poor, vascular invasion and no adjuvant chemotherapy were indicators of poor OS. Patients with LIPI intermediate/poor had worse OS especially among females and those with adjuvant chemotherapy (*p* < 0.05). Adjuvant chemotherapy related to better RFS and OS in patients with LIPI good (*p* < 0.05).

**Conclusions:**

Preoperative LIPI intermediate/poor can be an indicator of poor prognosis in patients with PDAC undergoing radical resection. LIPI good could be an effective marker of benefit from adjuvant chemotherapy. Larger studies are warranted for further validation.

## Introduction

Pancreatic ductal adenocarcinoma (PDAC) is one of the most lethal malignancies, which is predicted to be the second cause of cancer-associated mortality in the United States by 2030 (1). Most patients with PDAC have distant metastases or locally advanced disease at initial diagnosis, and less than 20% are eligible for resection. Radical resection remains the only potentially curative treatment for PDAC (2, 3). However, due to the highly aggressive nature of PDAC, many patients relapse within 1 year after radical resection. The median overall survival (OS) was 2 years for PDAC patients with stage I/II, and less than 1 year for those with stage III (4, 5). In addition, due to the high incidence of complications (up to 40%) after pancreatectomy and postoperative mortality (3%–5%) (2), appropriate patient selection is a key factor in the outcome of early-stage PDAC. Therefore, it is crucial to identify potential biomarkers that select PDAC patients who would benefit from radical resection and help make subsequent treatment decisions.

Serum lactate dehydrogenase (LDH) elevation has been identified as an indicator of poor prognosis in unresectable pancreatic cancer (6–8). A meta-analysis containing 76 studies showed that higher LDH levels (>245 U/L) were associated with unfavorable OS and PFS in a variety of solid tumors (9). LDH regulates the final step of glycolysis process to provides energy and biosynthesis for tumor cells (10). Enhanced LDH promotes tumor survival by inhibiting apoptosis, preventing necrosis in anoxic environment, and protecting tumor from reactive oxygen species (ROS) damage. LDH also facilitate tumor metastasis and angiogenesis by activating VEGF signaling pathway (11). What's more, LDH can promote immunosuppressive cells activation by increasing lactate production (12). Systemic inflammation indicators have been reported to be associated with tumors prognosis. Notably, derived neutrophil-to-lymphocyte ratio [dNLR, absolute neutrophil count/(absolute leukocyte count−absolute neutrophil count)] has been reported to predict prognosis in a variety of malignancies including melanoma (13), breast cancer (14), urothelial bladder cancer (15), renal cell carcinoma (16) and pancreatic cancer (17). dNLR reflects the composition of tumor microenvironment and determines the anti-tumor immune status. Neutrophils participate in suppressing immunity and facilitating tumor proliferation by inhibiting lymphokine activation (18), while lymphocytes participate in enhancing immunity and inhibiting tumor progression *via* cytotoxic cell and cytokine production (19). Therefore, the value of dNLR is closely related to tumor prognosis.

The lung immune prognostic index (LIPI), which combines dNLR and LDH, was first proposed by Mezquita et al. in 2018 (20) and predicts the prognosis of advanced non-small cell lung cancer (NSCLC) patients treated with immunotherapy. Since then, more and more studies have shown that LIPI was predictive of treatment outcomes in NSCLC and small cell lung cancer (21–25). More recently, there has been growing interest in extra-pulmonary cancer. LIPI has been reported to be predictive of the benefit of immunotherapy in other solid tumors (26, 27). It was also related to the prognosis of patients with osteosarcoma receiving standard treatment (28), esophageal squamous cell carcinoma undergoing curative surgery (29) or chemoradiotherapy (30), urothelial bladder cancer receiving radical cystectomy (31), and advanced breast cancer patients treated with trastuzumab emtansine (14). However, the value of LIPI in the prognosis of PDAC patients undergoing radical resection remains unknown. Therefore, this study aims to investigate the prognostic significance of preoperative LIPI in PDAC patients undergoing radical resection, and further explore whether LIPI affects treatment response to postoperative chemotherapy.

## Methods

### Study population

A total of 336 patients with pancreatic cancer who received resection at the Chinese People's Liberation Army (PLA) general hospital between February 2019 to April 2021 were included. The inclusion criteria were as follows: (1) diagnosed with stage I–III pancreatic cancer; (2) received radical resection and postoperative pathology histologically confirmed pancreatic ductal adenocarcinoma. The exclusion criteria were: (1) absence of preoperative blood test results; (2) loss to follow-up; (3) received preoperative chemotherapy or other treatments; (4) suffered from other malignancies, inflammatory diseases, autoimmune diseases, or trauma and (5) imaging examination revealed distant metastasis within two weeks after surgery. Following the above criteria, 205 patients were eventually enrolled in our study. Clinical characteristics as well as preoperative blood test results were recorded. Clinical characteristics included age, gender, tumor location, differentiation, tumor size, lymph node metastasis, TNM stage, neural invasion, vascular invasion, smoking history, drinking history and postoperative adjuvant chemotherapy. Preoperative blood test results included ratio of neutrophil to white blood cell, LDH, CEA, CA-125 and CA19-9 levels. All patients were followed up through electronic medical records and telephone consultations until March 31, 2022. This study was approved by the Ethics Committee of Chinese PLA General Hospital and was conducted according to the principles of the Declaration of Helsinki.

### Assessment

Blood tests results were collected within one week before surgery. LIPI was defined as dNLR (equal to the ratio of neutrophils to white blood cells divided by 1 min the ratio of neutrophils to white blood cells) and LDH. The cutoff values of dNLR and LDH were calculated by X-tile software based on overall survival (32), which were 1.4 and 225 U/L, respectively. Based on preoperative dNLR and LDH levels, patients were divided into three groups: LIPI good group with dNLR < 1.4 and LDH < 225 U/L, LIPI intermediate group with dNLR < 1.4 and LDH ≥ 225 U/L, or dNLR ≥ 1.4 and LDH < 225 U/L, LIPI poor group with dNLR ≥ 1.4 and LDH ≥ 225 U/L. LIPI intermediate and poor group were integrated for prognostic analysis. Overall survival (OS) was defined as the time from the date of surgery to the date of death or the date of last follow-up. Recurrence-free survival (RFS) was defined as the time from the date of surgery to the first recurrence or the last follow-up. Recurrence included local recurrence, lymph node metastasis and distant metastasis to liver, lung or other sites. The pathological stage was determined according to the eighth edition of the TNM classification.

### Statistical analysis

The optimal cut-off value of dNLR and LDH were identified by X-tile 3.6.1 (Yale University, New Haven, CT, United States). Clinical characteristics were presented as categorical variables and compared using the Chi-square test. Kaplan-Meier method was used to analyze survival data and the significance analysis was implemented by log-rank test. Univariate and multivariate analyses were performed by Cox proportional hazards models to assess the independent prognostic value of preoperative LIPI and other factors. Calibration plots and C-index were used to evaluate the calibration and discrimination of Cox proportional hazards models. Subgroup analysis and *p*-value for interaction were computed to identify specific population who might benefit from this treatment. IBM SPSS 26.0 (SPSS Inc., Chicago, IL, United States) and GraphPad Prism 8 (La Jolla, CA, United States) were used for survival analyses. R software (v4.1.2, R Foundation for Statistical Computing, Vienna, Austria) and R studio (v1.4.1103, Integrated Development for R, Boston, United States) were used for internal validation. All statistical tests were two-sided tests with *p* < 0.05 denoted statistical significance.

## Results

### Patients' characteristics

A total of 205 PDAC patients receiving radical resection with complete clinical and survival data were analyzed (Supplementary Figure S5). Surgical methods included laparotomy, laparoscopic surgery and robot-assisted surgery. Chemotherapy regimens included AS (nab-paclitaxel 125 mg/m^2^ on day 1 and day 8 plus S-1 40–60 mg twice daily on day 1 to 14 of each cycle, 3 weekly scheme), GS (gemcitabine 1,000 mg/m^2^ on day 1 and day 8 plus S-1 40–60 mg twice daily on day 1 to 14 of each cycle, 3 weekly scheme), AG (nab-paclitaxel 125 mg/m^2^ plus gemcitabine 1,000 mg/m^2^ on day 1 and day 8 of each cycle, 3 weekly scheme), and nab-paclitaxel or S-1 as a single agent according to the patient's physical state. Clinical characteristics are summarized in Table 1. Among the 205 patients, the median age at diagnosis was 63 years old (range from 31 to 82), 62.4% were males, 23.9% with elevated CEA, 15.6% with elevated CA-125, 67.8% with elevated CA19-9, 60.0% located in the head of pancreas, 38.5% had a smoking history and 43.3% had a drinking history. Of the patients, 50.2% were well or moderately differentiated adenocarcinoma, 74.1% had neural invasion, 21.5% had vascular invasion, 52.2%, 39.0%, and 8.8% were stage I, stage II, and stage III, respectively; 70.2% received adjuvant chemotherapy, 40.5% with dNLR < 1.4, 86.3% with LDH < 225 U/L. Patients in the LIPI good, intermediate, and poor group were 36.6%, 53.7%, and 9.8%, respectively.

**Table 1:** Characteristics of patients with resected PDAC.

Characteristics	No. of patients (*n* = 205)	Percentage (%)
Age (year), median (range)	63 (31–82)	
<63	102	49.8
≥63	103	50.2
Gender		
Male	128	62.4
Female	77	37.6
CEA (μg/L)		
≤5	156	76.1
>5	49	23.9
CA-125 (U/mL)		
≤35	173	84.4
>35	32	15.6
CA19-9 (U/mL)		
≤37	66	32.2
>37	139	67.8
Tumor location		
Head	123	60.0
Body/tail	82	40.0
Differentiation		
Well/moderately	103	50.2
Poorly	102	49.8
TNM stage		
I	107	52.2
II	80	39.0
III	18	8.8
Neural invasion		
No	53	25.9
Yes	152	74.1
Vascular invasion		
No	161	78.5
Yes	44	21.5
Adjuvant chemotherapy		
No	61	29.8
Yes	144	70.2
Smoking history		
No	126	61.5
Yes	79	38.5
Drinking history		
No	116	56.6
Yes	89	43.3
dNLR		
<1.4	83	40.5
≥1.4	122	59.5
LDH (U/L)		
<225	177	86.3
≥225	28	13.7
LIPI		
Good	75	36.6
Intermediate	110	53.7
Poor	20	9.8

### LIPI intermediate/poor associated with worse RFS and os

**Figure 1:**
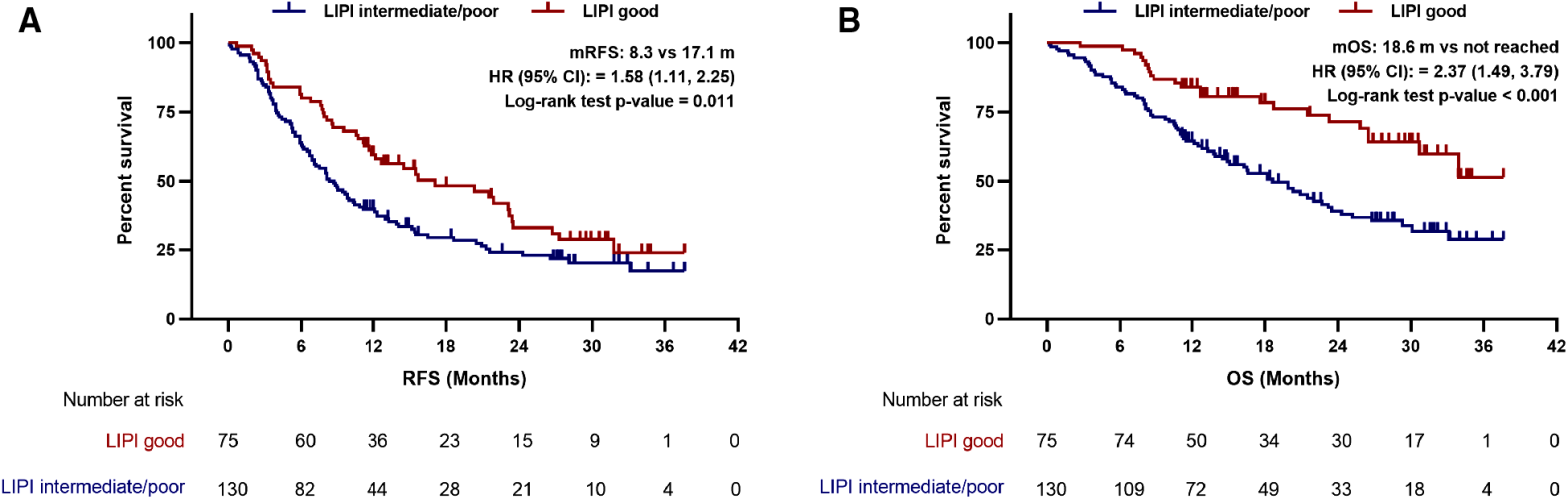
Preoperative LIPI associated with (**A**) recurrence-free survival and (**B**) overall survival in PDAC patients undergoing radical resection. LIPI, lung immune prognostic index; mRFS, median recurrence-free survival; mOS, median overall survival; HR, hazard ratio; CI, confidence interval.

The median follow-up time was 28.2 months (range, 0.2–36.7 months). At the time of last follow-up, 70.2% of the patients relapsed and 48.3% died, the median RFS and OS was 10.8 and 24.3 months, respectively. In the LIPI intermediate/poor and LIPI good group, the median RFS was 8.3 and 17.1 months (HR: 1.58; 95%CI: 1.11, 2.25; *p* = 0.011), respectively (Figure 1A). The median OS was 18.6 months and not reached (HR: 2.37; 95%CI: 1.49, 3.79; *p* < 0.001) in the LIPI intermediate/poor and LIPI good group, respectively (Figure 1B). The RFS and OS of LIPI intermediate/poor group were significantly worse than that of LIPI good group.

### LIPI was an independent prognostic factors for os

Univariate Cox regression indicated that increased CA-125 level, poorly differentiation, stage II/III, vascular invasion and LIPI intermediate/ poor were related to worse RFS in PDAC patients receiving radical resection (*p* < 0.05). Multivariate Cox regression revealed that only CA-125 > 35 (HR: 1.48; 95%CI: 1.02, 2.44; *p* = 0.041) and vascular invasion (HR: 1.62; 95%CI: 1.08, 2.43; *p* = 0.020) were independent risk factors for poor RFS (Table 2). Univariate Cox regression for OS was similar with the outcome of RFS that increased CA-125 level, poorly differentiation, stage II/III, vascular invasion, LIPI intermediate/poor and no chemotherapy were related to worse OS (*p* < 0.05). After multivariate Cox regression, vascular invasion (HR: 1.88; 95%CI: 1.19, 2.97; *p* = 0.007), chemotherapy (HR:0.52; 95%CI: 0.34, 0.79; *p* = 0.002) and LIPI intermediate/poor (HR:1.69; 95%CI: 1.01, 2.80; *p* = 0.044) were independent prognostic factors for OS (Table 3). The C-index of multivariate Cox regression model for RFS and OS for were 0.650 (0.603, 0.697) and 0.710 (0.663, 0.757), respectively. Calibration plots showed good performance of multivariate Cox regression model for RFS and OS (Supplementary Figures S1–S4).

**Table 2:** Univariate and multivariate analysis for RFS in patients with resected PDAC.

Variables	Category	Univariate analysis	Multivariate analysis
		HR (95% CI)	*p*-value	HR (95% CI)	*p*-value
Age	≥63 vs. <63	1.16 (0.83, 1.61)	0.383	–	–
Gender	female vs. male	1.01 (0.72, 1.43)	0.936	–	–
CEA	>5 vs. ≤5	1.16 (0.80, 1.69)	0.437	–	–
CA-125	>35 vs. ≤35	1.94 (1.27, 2.96)	0.002	1.48 (1.02, 2.44)	**0.041**
CA19-9	>37 vs. ≤37	1.08 (0.76, 1.54)	0.665	–	–
Tumor location	body/tail vs. head	1.01 (0.72, 1.41)	0.977	–	–
Differentiation	poorly vs. well/moderately	1.67 (1.20, 2.33)	0.003	1.31 (0.92, 1.88)	0.134
TNM stage	stage II/III vs. stage I	1.62 (1.17, 2.25)	0.004	1.20 (0.84, 1.73)	0.316
Neural invasion	yes vs. no	1.29 (0.88, 1.91)	0.194	–	–
Vascular invasion	yes vs. no	2.01 (1.39, 2.91)	<0.001	1.62 (1.08, 2.43)	**0.020**
Smoking history	yes vs. no	0.90 (0.65, 1.27)	0.558	–	–
Drinking history	yes vs. no	0.98 (0.71, 1.37)	0.917	–	–
Chemotherapy	yes vs. no	0.76 (0.53, 1.08)	0.123	–	–
LIPI	intermediate/poor vs. good	1.58 (1.11, 2.25)	0.011	1.39 (0.97, 2.00)	0.076

**Table 3:** Univariate and multivariate analysis for OS in patients with resected PDAC.

Variables	Category	Univariate analysis	Multivariate analysis
		HR (95% CI)	*p*-value	HR (95% CI)	*p*-value
Age	≥63 vs. <63	1.41 (0.94, 2.09)	0.093	–	–
Gender	female vs. male	1.29 (0.86, 1.93)	0.218	–	–
CEA	>5 vs. ≤5	0.91 (0.56, 1.46)	0.688	–	–
CA-125	>35 vs. ≤35	1.86 (1.14, 3.04)	0.014	1.49 (0.88, 2.52)	0.136
CA19-9	>37 vs. ≤37	0.91 (0.60, 1.39)	0.672	–	–
Tumor location	body/tail vs. head	0.74 (0.49, 1.13)	0.164	–	–
Differentiation	poorly vs. well/moderately	1.62 (1.08, 2.41)	0.019	1.18 (0.76, 1.83)	0.455
TNM stage	stage II/III vs. stage I	1.74 (1.17, 2.60)	0.007	1.16 (0.74, 1.83)	0.520
Neural invasion	yes vs. no	1.09 (0.69, 1.73)	0.705	–	–
Vascular invasion	yes vs. no	2.17 (1.42, 3.31)	<0.001	1.88 (1.19, 2.97)	**0.007**
Smoking history	yes vs. no	0.91 (0.61, 1.37)	0.650	–	–
Drinking history	yes vs. no	0.86 (0.58, 1.28)	0.457	–	–
Chemotherapy	yes vs. no	0.46 (0.31, 0.69)	<0.001	0.52 (0.34, 0.79)	**0.002**
LIPI	intermediate/poor vs. good	2.37 (1.49, 3.79)	<0.001	1.69 (1.01, 2.80)	**0.044**

### Subgroup analysis

**Figure 2:**
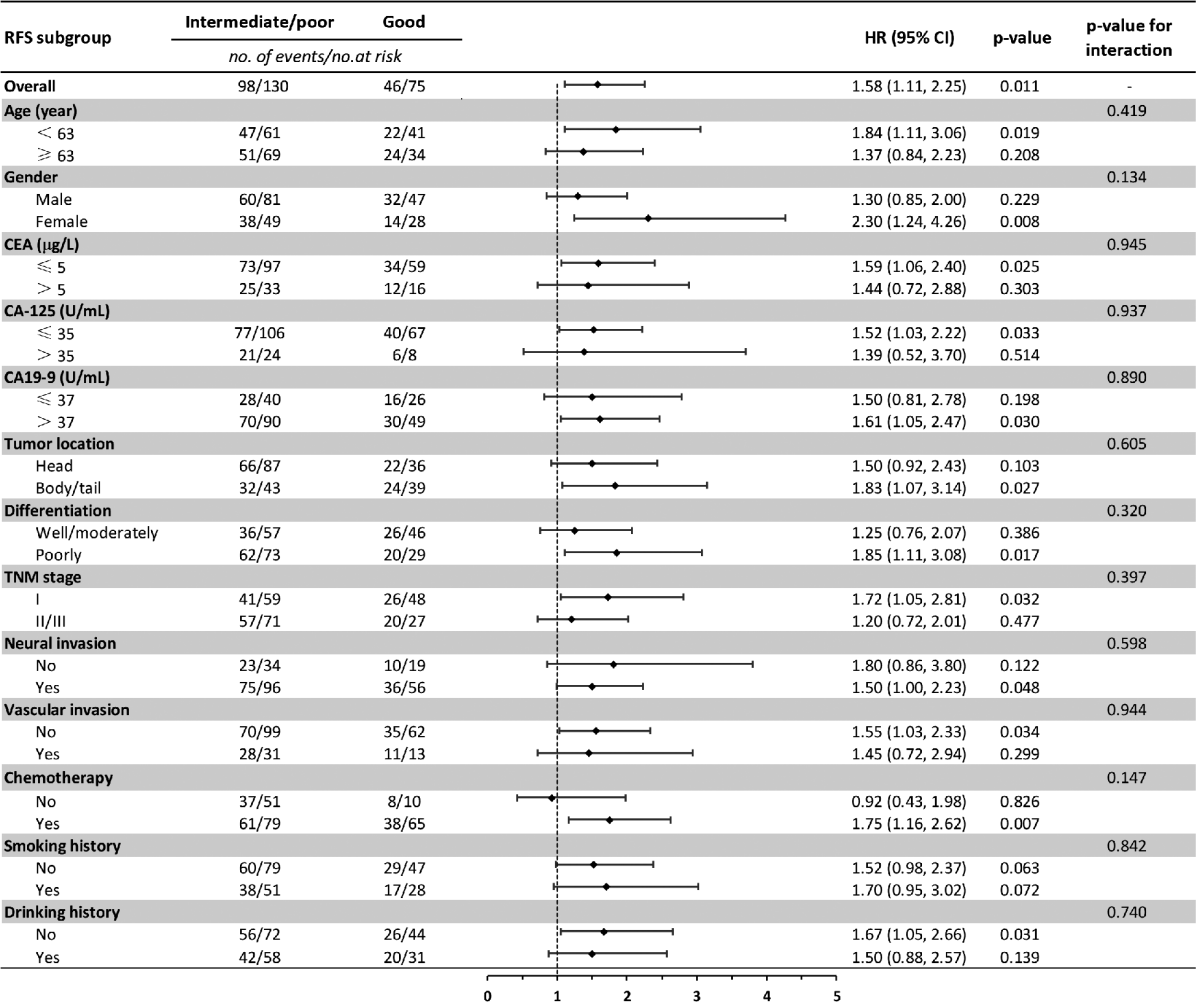
Subgroup analysis of the association between preoperative LIPI and recurrence-free survival. RFS, recurrence-free survival; HR, hazard ratio; CI, confidence interval.

**Figure 3:**
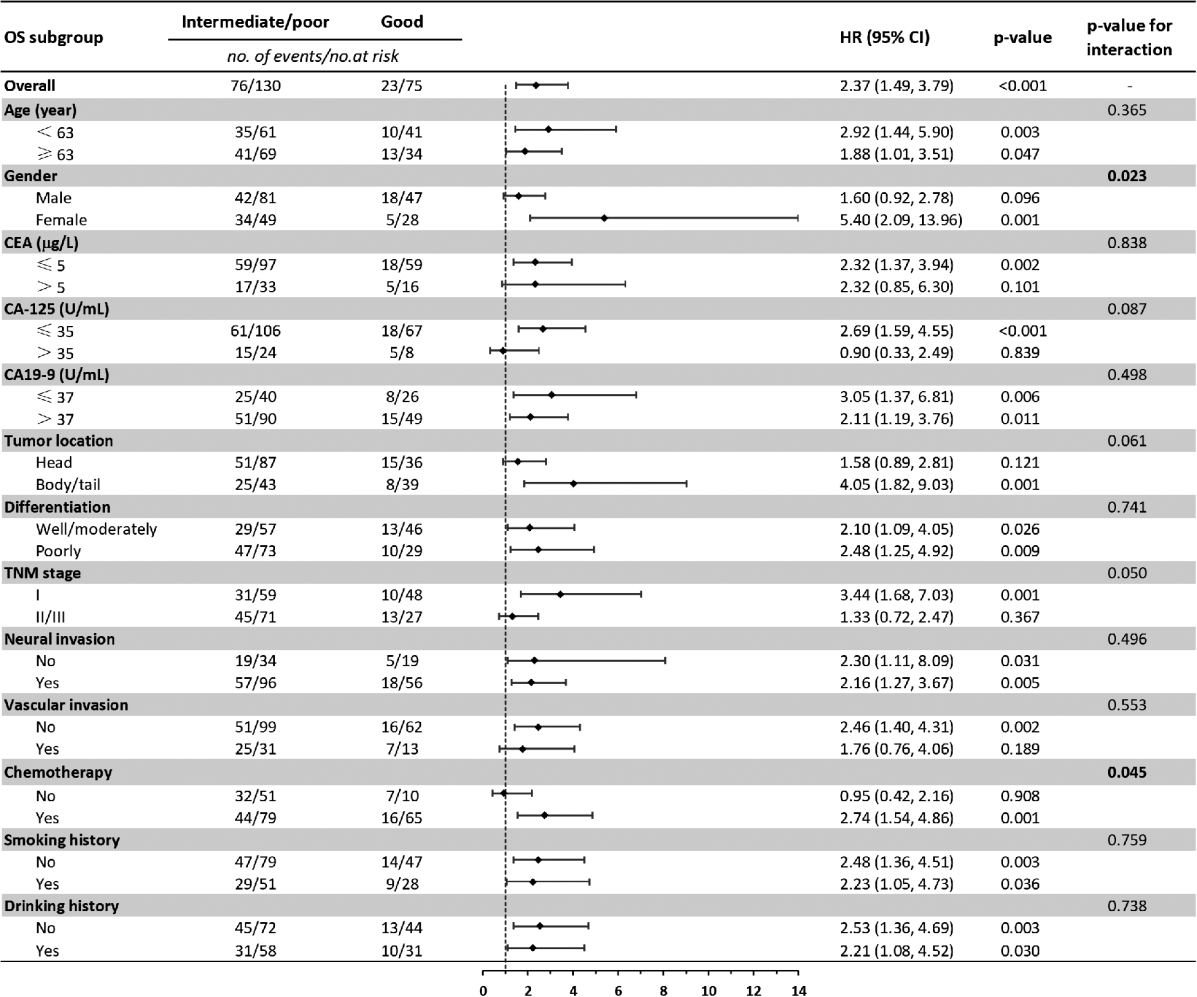
Subgroup analysis of the association between preoperative LIPI and overall survival. OS, overall survival; HR, hazard ratio; CI, confidence interval.

Baseline comparison between the LIPI good and LIPI intermediate/poor group showed that no differences were observed in age, gender, CEA, CA-125, CA19-9, neural invasion, vascular invasion, smoking and drinking history. Patients with LIPI intermediate/poor tended to have pancreatic head cancer (*p* = 0.008), poorly differentiation (*p* = 0.016), late TNM stage (*p* = 0.010) and less adjuvant chemotherapy (*p* < 0.001) (Table 4). We further conducted subgroup analysis stratified by these characteristics. The results showed that no significant differences were found between subgroups for RFS in LIPI good and LIPI intermediate/poor group (Figure 2). Patients with LIPI intermediate/poor had worse OS, especially among females and those with adjuvant chemotherapy (*p* < 0.05) (Figure 3).

**Table 4:** Baseline comparison between LIPI good and LIPI intermediate/poor.

Clinical characteristics	LIPI	*p*-value
	Good		Intermediate/poor
Age (year)			0.285
<63	41	61	
≥63	34	69	
Gender			0.959
Male	47	81	
Female	28	49	
CEA (μg/L)			0.512
≤5	59	97	
>5	16	33	
CA-125 (U/ml)			0.139
≤35	67	106	
>35	8	24	
CA19-9 (U/ml)			0.565
≤37	26	40	
>37	49	90	
Tumor location			**0** **.** **008**
Head	36	87	
Body/tail	39	43	
Differentiation			**0** **.** **016**
Well/moderately	46	57	
Poorly	29	73	
TNM stage			**0** **.** **010**
I	48	59	
II	25	55	
III	2	16	
Neural invasion			0.897
No	19	34	
Yes	56	96	
Vascular invasion			0.274
No	62	99	
Yes	13	31	
Adjuvant chemotherapy			**0** **.** **000**
No	10	51	
Yes	65	79	
Smoking history			0.788
No	47	79	
Yes	28	51	
Drinking history			0.648
No	44	72	
Yes	31	58	

### LIPI good benefited from adjuvant chemotherapy

**Figure 4:**
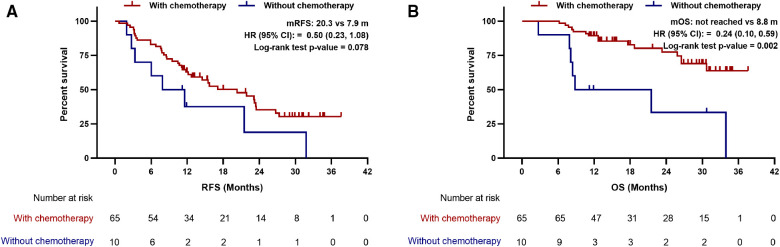
Association between postoperative chemotherapy with (**A**) recurrence-free survival and (**B**) overall survival in patients with LIPI good. mRFS, median recurrence-free survival; mOS, median overall survival; HR, hazard ratio; CI, confidence interval.

**Figure 5:**
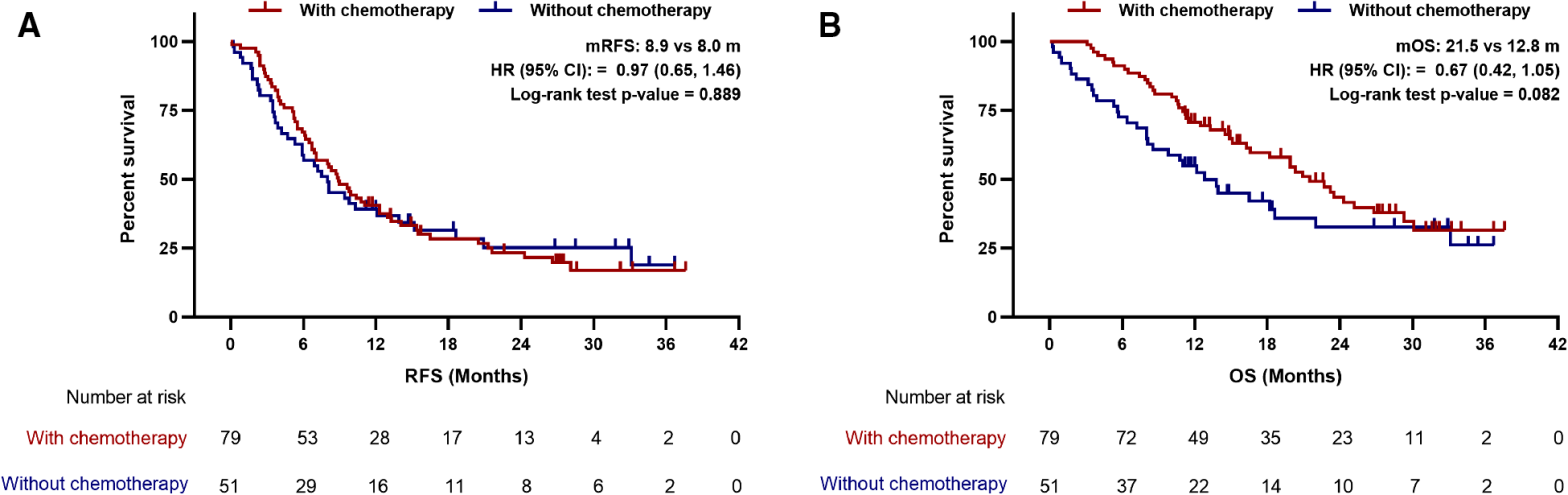
Association between postoperative chemotherapy with (**A**) recurrence-free survival and (**B**) overall survival in patients with LIPI intermediate/poor. mRFS, median recurrence-free survival; mOS, median overall survival; HR, hazard ratio; CI, confidence interval.

To further explore whether LIPI helps stratifying resected PDAC patients who may benefit from postoperative chemotherapy. Kaplan-Meier curves and multivariate Cox regression analyses were performed to compare the survival of patients treated with and without adjuvant chemotherapy in different LIPI groups. Interestingly, the RFS and OS of patients with adjuvant chemotherapy are better than that of patients without adjuvant chemotherapy in LIPI good group (median RFS: 20.3 vs. 7.9 m, *p* = 0.078; median OS: not reached vs. 8.8 m, *p* = 0.002) (Figure 4). After adjusting for CA-125, differentiation, TNM stage and vascular invasion, adjuvant chemotherapy was an independent prognostic factor for good RFS (HR:0.32; 95%CI: 0.14, 0.75; *p* = 0.008) and OS (HR:0.08; 95%CI: 0.02, 0.25; *p* < 0.001) in the LIPI good group (Supplementary Table S1). However, no differences were observed in OS and RFS between patients with and without adjuvant chemotherapy in LIPI intermediate/poor group (*p* > 0.05) (Figure 5, Supplementary Table S2).

## Discussion

Although radical resection offers the only hope of curing PDAC, not all patients benefit from this treatment. Thus, the identification of potential biomarkers that predict population benefits from radical resection and subsequent treatment is needed. Peripheral inflammation indicators are generally easy to acquire in clinical practice. Previous studies have found that inflammatory markers are associated with the prognosis of patients with pancreatic cancer (33–38). The dNLR, defined as dividing neutrophils by leukocytes minus neutrophils, has been reported to be a promising independent prognostic indicator in a single-center, large cohort and full-stage PDAC patients (17). Suzuki et al. also found that pretreatment dNLR was an important biomarker in stratifying unresectable PDAC patients who may benefit from gemcitabine (39). Meanwhile, the prognostic role of pretreatment LDH levels has been well demonstrated in pancreatic cancer (40–43). Xiao et al. reported that among patients with advanced pancreatic cancer receiving chemotherapy, those with elevated LDH had a 2.47-fold increased risk of death, but no significant differences were found between elevated LDH and normal LDH group in patients who did not receive chemotherapy (44). Therefore, dNLR and LDH are both useful prognostic markers of pancreatic cancer. The combination of dNLR and LDH, also known as LIPI, was first revealed as a prognostic factor for advanced NSCLC patients treated with immune checkpoint inhibitors (20). More and more studies indicated that LIPI was associated with therapeutic outcomes in many malignancies (14, 21–31). However, there is a lack of researches exploring the value of preoperative LIPI in the outcomes of resectable PDAC patients treated with radical surgery.

To our knowledge, this is the first study to elucidate the relationship between preoperative LIPI status and survival outcomes of PDAC patients undergoing radical surgery. In this study, the median RFS and OS was 10.8 and 24.3 months, respectively, which were consistent with previous literature reports (4). Due to the small sample size of patients with LIPI poor, LIPI poor group was incorporated into LIPI intermediate group for survival analyses. Our results showed that preoperative LIPI intermediate/poor was associated with worse RFS and OS in PDAC patients treated with radical surgery. Univariate and multivariate Cox regression indicated that preoperative LIPI intermediate/poor was an independent indicator of poor OS but not RFS, which was consistent with the results of a recently published research of SCLC patients treated with immunotherapy plus chemotherapy (24). Nevertheless, the negative findings of RFS should be viewed carefully due to its retrospective nature. Multiple factors affect RFS, such as the methods, frequency, and physician's evaluation of postoperative examination. In contrast, the significant difference in OS between the two groups was more credible.

Inflammation predisposes to the progression of all stages of cancer, including PDAC. Increased dNLR is mainly associated with neutrophilia. Neutrophils are the main inflammatory cells involved in tumor-promoting. In the process of metastasis, neutrophils can form complexes with cancer cells and aid in the adhesion and translocation of metastatic seeds to the vessel wall. The neutrophil-cancer cells complexes can also protect these metastatic seeds from immune surveillance at their most vulnerable moments away from established immunosuppressive microenvironment of the primary tumor (45). LDH is an important enzyme in final step of glycolysis process, which provides energy and biosynthesis for tumor cells. Tumor burden can be reflected by LDH levels as rapidly growing tumors can produce large amounts of LDH. Increased LDH can also promote tumor progression by inhibiting tumor cell apoptosis, activating VEGF pathway, and immunosuppressive cells activation (10–12). LIPI is determined by dNLR and LDH. The above evidence well elucidated the underlying mechanism of the association between LIPI and prognosis of PDAC patients.

We also found that preoperative LIPI was associated with tumor location, tumor differentiation, TNM stage, and adjuvant chemotherapy. Similar results have been reported in the systemic inflammation and nutrition status of resected PDAC (46). Patients with tumor located in the head of pancreas, poorly differentiation, advanced TNM stage or without adjuvant chemotherapy were tend to have intermediate/poor LIPI. Pancreatic head cancer usually leads to biliary and digestive tract obstruction as well as pancreatitis, which causes elevated levels of inflammatory indicators, such as neutrophils, and eventually lead to elevated dNLR. It is well known that poor tumor differentiation, advanced TNM stage and no chemotherapy are all recognized indicators of poor prognosis for PDAC, but the reason why they are related to LIPI is still unclear. However, among these factors correlated to LIPI, only adjuvant chemotherapy was found as an independent prognostic factor for OS. We further performed a subgroup analysis stratifying by clinical characteristics and the results showed that patients with LIPI intermediate/poor had worse OS than those with LIPI good, especially among females and those receiving adjuvant chemotherapy, which indicated that for the patients with LIPI intermediate/poor, adjuvant chemotherapy might not be the optimal postoperative treatment. In addition, the result that LIPI good patients treated with adjuvant chemotherapy had better RFS and OS than those without adjuvant chemotherapy further confirmed preoperative LIPI as an effective biomarker in selecting resected PDAC patients who may benefit from adjuvant chemotherapy. However, further studies are needed to verify these results.

Our study had several limitations. Firstly, it was a retrospective study conducted at a single center with a limited number of participants and potentially incomplete information. Thus, residual confounding factors and selection bias are inevitable. However, we adjusted as many possible confounders as we could and participants are enrolled consecutively in strict accordance with our inclusion and exclusion criteria. Data from external medical centers are being collected and the results will be presented in subsequent studies. Secondly, due to the COVID-19 pandemic, many patients failed to complete a full course of postoperative chemotherapy and regularly comprehensive imaging reviews. Therefore, the results of our study should be interpreted carefully. Finally, the dNLR and LDH cutoff values were calculated by X-tile software based on the data collected in our center, which may not be optimal. We also performed Cox regression analyses with dNLR and LDH as continuous variables, and the results remained stable (Supplementary Table S3). Despite the deficiencies in this study, it provided an easy and non-invasive way for identifying PDAC patients who might benefit from radical resection and subsequent adjuvant chemotherapy in clinical practice.

## Conclusion

Our study revealed the prognostic value of preoperative LIPI in patients with PDAC treated with radical resection. Preoperative LIPI intermediate/poor can be an indicator of poor prognosis in patients with PDAC undergoing radical resection. LIPI good could be an effective marker of benefit from adjuvant chemotherapy. Larger studies are warranted for further validation.

## Data Availability

The original contributions presented in the study are included in the article/**Supplementary Material**, further inquiries can be directed to the corresponding authors.
