# Supplementary material for: Preoperative lung immune prognostic index predicts survival in patients with pancreatic cancer undergoing radical resection
Source: Front Surg. 2023 Jan 6;9:1002075. doi: 10.3389/fsurg.2022.1002075 (PMC9852768; doi:10.3389/fsurg.2022.1002075)
Supplement: Supplementary file 1 [file Table1.docx]

Table S1. Cox regression analyses of ACT associated with OS and RFS in LIPI good group

| **Category** | **Univariate analysis** | |  | **Multivariate analysis** | |
| --- | --- | --- | --- | --- | --- |
|  | **Crude HR (95%CI)** | **p-value** |  | **Adjusted HR (95%CI)** | **p-value** |
| **OS** |  |  |  |  |  |
| No ACT | 1(Ref) |  |  | 1(Ref) |  |
| with ACT | 0.24 (0.10~0.59) | 0.002 |  | 0.08 (0.02~0.25) | <0.001 |
| **RFS** |  |  |  |  |  |
| No ACT | 1(Ref) |  |  | 1(Ref) |  |
| with ACT | 0.50 (0.23~1.08) | 0.078 |  | 0.32 (0.14~0.75) | 0.008 |

Multivariate analysis adjusted for CA-125, differentiation, TNM stage, and vascular invasion.

Abbreviations: ACT, adjuvant chemotherapy; OS, overall survival; RFS, recurrence-free survival; LIPI, lung immune prognostic index; HR, hazard ratio; CI, confidence interval; Ref, reference.

Table S2. Cox regression analyses of ACT associated with OS and RFS in LIPI intermediate/poor group

| **Category** | **Univariate analysis** | |  | **Multivariate analysis** | |
| --- | --- | --- | --- | --- | --- |
|  | **Crude HR (95%CI)** | **p-value** |  | **Adjusted HR (95%CI)** | **p-value** |
| **OS** |  |  |  |  |  |
| No ACT | 1(Ref) |  |  | 1(Ref) |  |
| with ACT | 0.67 (0.42~1.05) | 0.082 |  | 0.64 (0.41~1.02) | 0.062 |
| **RFS** |  |  |  |  |  |
| No ACT | 1(Ref) |  |  | 1(Ref) |  |
| with ACT | 0.97 (0.64~1.46) | 0.889 |  | 0.96 (0.64~1.46) | 0.864 |

Multivariate analysis adjusted for CA-125, differentiation, TNM stage, and vascular invasion.

Abbreviations: ACT, adjuvant chemotherapy; OS, overall survival; RFS, recurrence-free survival; LIPI, lung immune prognostic index; HR, hazard ratio; CI, confidence interval; Ref, reference.

Table S3. Crude and adjusted HRs and 95%CI of dNLR and LDH associated with OS and RFS

| **Variable** | **Univariate analysis** | |  | **Multivariate analysis** | |
| --- | --- | --- | --- | --- | --- |
|  | **Crude HR (95%CI)** | **p-value** |  | **Adjusted HR (95%CI)** | **p-value** |
| **OS** |  |  |  |  |  |
| dNLR continuous | 1.54 (1.32~1.8) | <0.001 |  | 1.53 (1.29~1.81) | <0.001 |
| dNLR < 1.4 | 1(Ref) |  |  | 1(Ref) |  |
| dNLR ≥ 1.4 | 2.02 (1.31~3.12) | 0.001 |  | 1.48 (0.93~2.36) | 0.099 |
| LDH (Per 10 U/L) | 1.12 (1.07~1.16) | <0.001 |  | 1.10 (1.06~1.15) | <0.001 |
| LDH < 225 | 1(Ref) |  |  | 1(Ref) |  |
| LDH ≥ 225 | 5.02 (3.18~7.91) | <0.001 |  | 3.71 (2.27~6.09) | <0.001 |
| **RFS** |  |  |  |  |  |
| dNLR continuous | 1.40 (1.19~1.65) | <0.001 |  | 1.34 (1.12~1.60) | 0.001 |
| dNLR < 1.4 | 1(Ref) |  |  | 1(Ref) |  |
| dNLR ≥ 1.4 | 1.49 (1.06~2.10) | 0.022 |  | 1.22 (0.84~1.76) | 0.294 |
| LDH (Per 10 U/L) | 1.06 (1.02~1.11) | 0.002 |  | 1.05 (1.02~1.09) | 0.006 |
| LDH < 225 | 1(Ref) |  |  | 1(Ref) |  |
| LDH ≥ 225 | 3.13 (2.00~4.88) | <0.001 |  | 2.69 (1.67~4.32) | <0.001 |

Multivariate analysis adjusted for CA-125, differentiation, TNM stage, vascular invasion and adjuvant chemotherapy.

Abbreviations: HR, hazard ratio; CI, confidence interval; dNLR, derived neutrophil-to- lymphocyte ratio; LDH, lactate dehydrogenase; OS, overall survival; RFS, recurrence-free survival; Ref, reference.
