# Supplementary figures and images for: Preoperative lung immune prognostic index predicts survival in patients with pancreatic cancer undergoing radical resection
Source: Front Surg. 2023 Jan 6;9:1002075. doi: 10.3389/fsurg.2022.1002075 (PMC9852768; doi:10.3389/fsurg.2022.1002075)

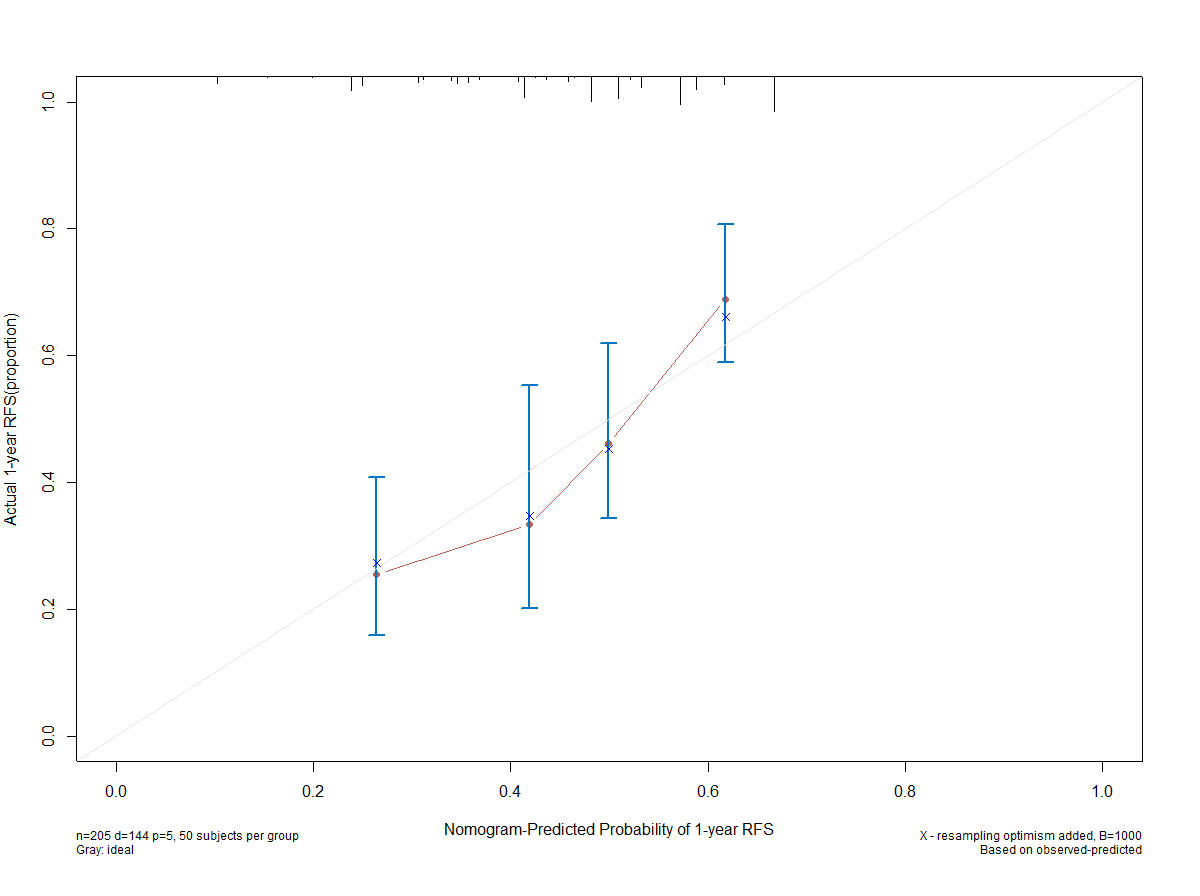

Supplement: Supplementary file 2 [file Image1.png]

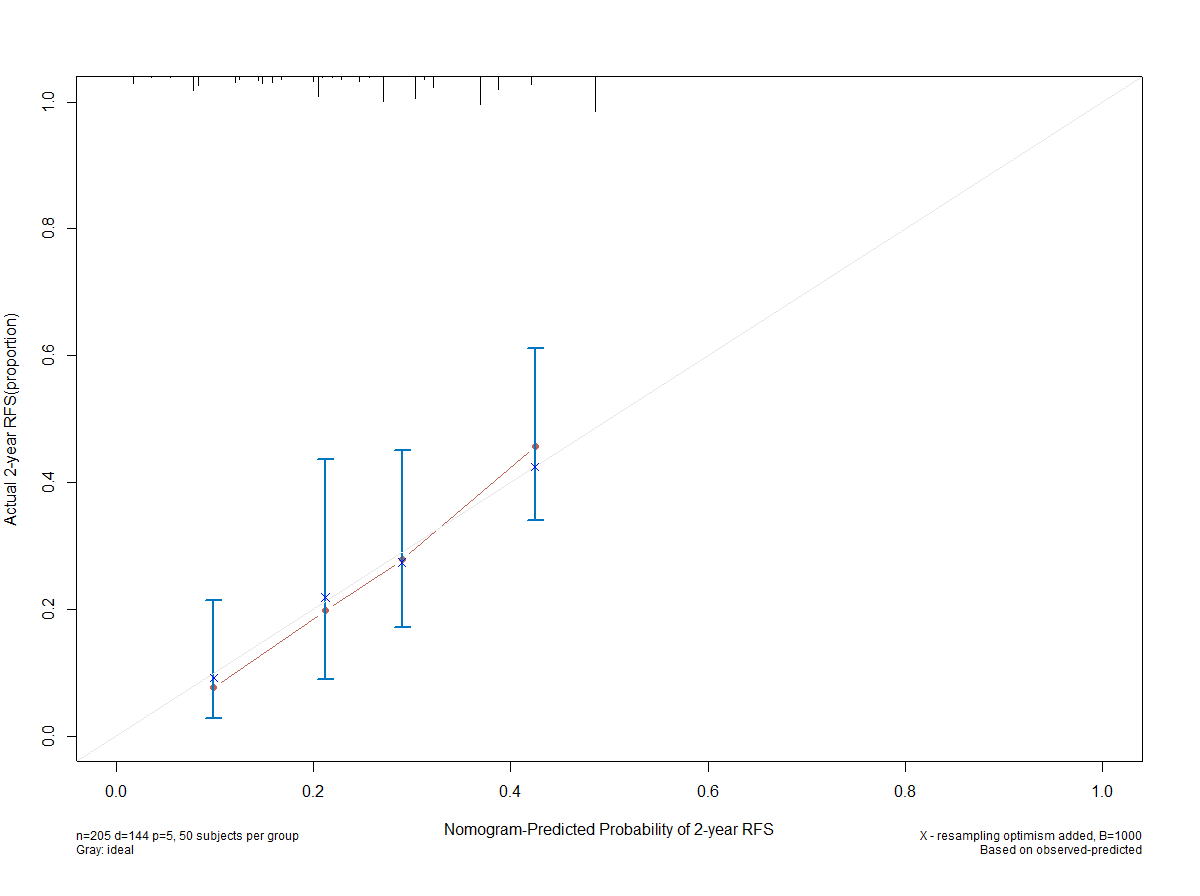

Supplement: Supplementary file 3 [file Image2.png]

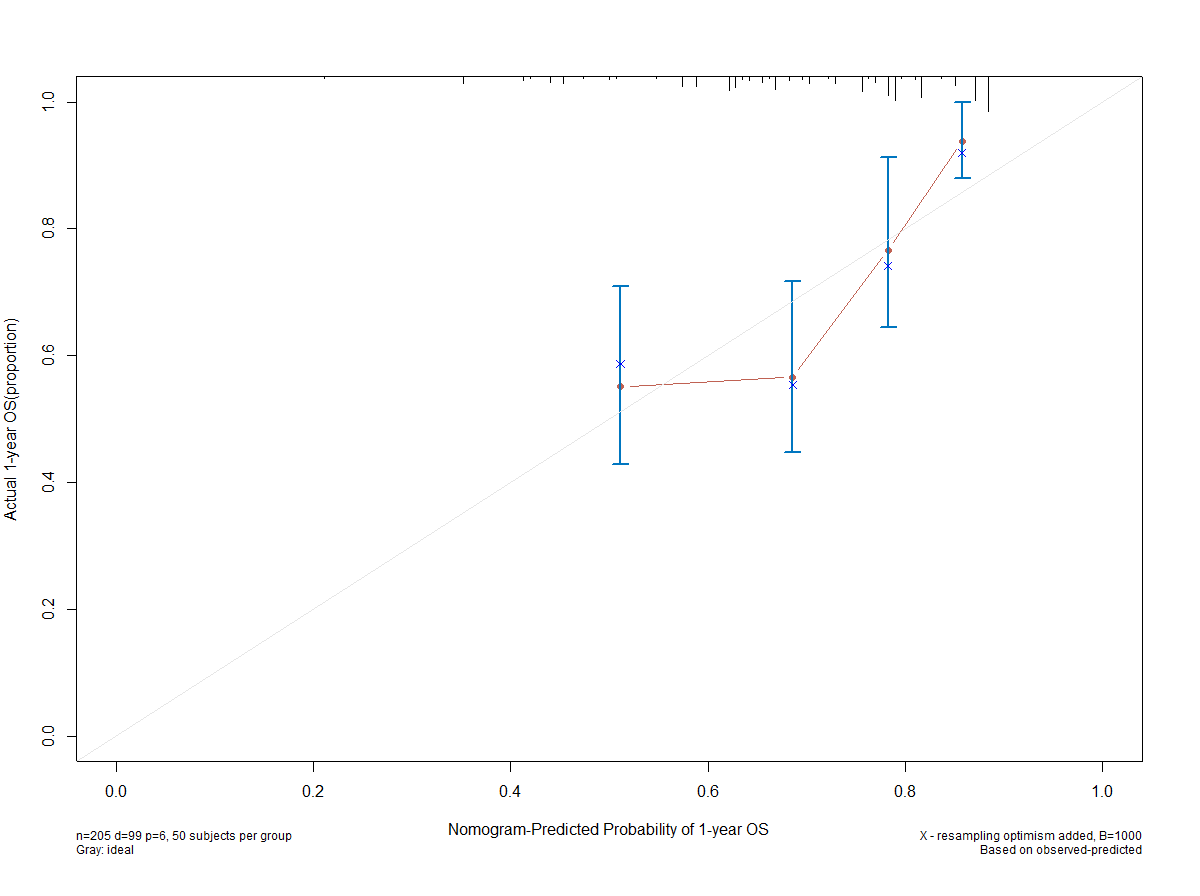

Supplement: Supplementary file 4 [file Image3.png]

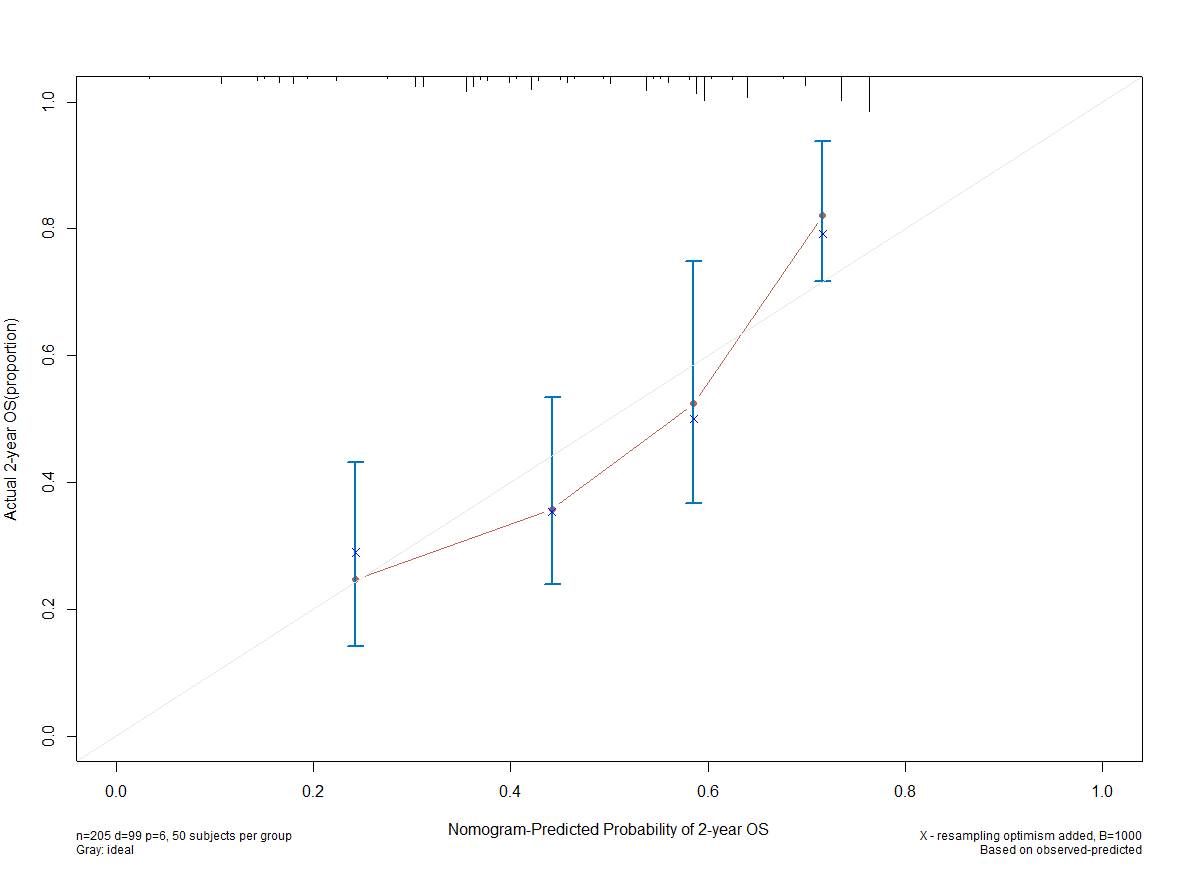

Supplement: Supplementary file 5 [file Image4.png]

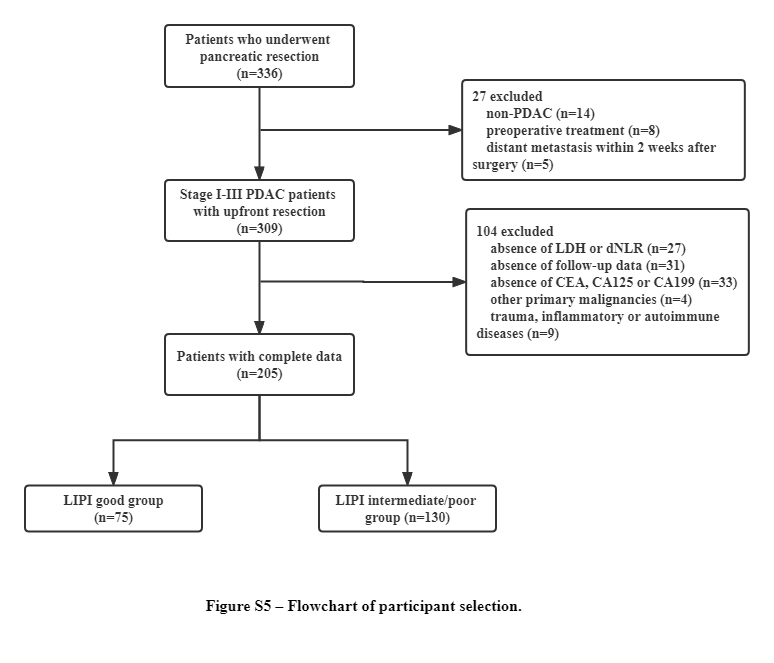

Supplement: Supplementary file 6 [file Image5.png]
